# Analysis of Consensus Molecular Subtypes of Colorectal Cancer in Oman with Clinicopathologic Correlation

**DOI:** 10.3390/ijms27042038

**Published:** 2026-02-21

**Authors:** Shaista Rehman, Asem Shalaby, Said A. Al-Busafi, Moon Fai Chan, Shaima Al Khabouri, Mustafa Al Hinai, Adhari Al Zaabi, Mohammad Al Masqari, Asim Qureshi, Mohammed Al-Azri

**Affiliations:** 1 Department of Family Medicine and Public Health, College of Medicine and Health Sciences, Sultan Qaboos University, Muscat 123, Oman; 2 Department of Pathology, College of Medicine and Health Sciences, Sultan Qaboos University, Muscat 123, Oman; 3 Department of Pathology, College of Medicine, Mansoura University, Mansoura 1156, Egypt; 4 Gastroenterology and Hepatology Unit, Department of Medicine, College of Medicine and Health Sciences, Sultan Qaboos University, Muscat 123, Oman; 5 Medical Research Center, Sultan Qaboos University, Muscat 123, Oman; 6 Department of Medicine, Faculty of Medicine and Health, Sepuluh Nopember Institute of Technology, Surabaya 60111, Indonesia; 7 Department of Biochemistry, College of Medicine and Health Sciences, Sultan Qaboos University, Muscat 123, Oman; 8 Human and Clinical Anatomy Department, College of Medicine and Health Sciences, Sultan Qaboos University, Muscat 123, Oman; 9 Department of Pathology, The Royal Hospital, Ministry of Health, Muscat 113, Oman

**Keywords:** colorectal cancer, consensus molecular subtypes, immunohistochemistry, molecular testing, Oman

## Abstract

Colorectal cancer (CRC) is a major public health challenge in Oman and a leading cause of cancer-related mortality. The rising incidence has been associated with lifestyle changes, urbanization, and genetic factors. The CRC Subtyping Consortium has defined four consensus molecular subtypes (CMS1–CMS4); however, data on their distribution in the Gulf region, including Oman, remain limited. This study aimed to characterize the distribution of CMS subtypes in Omani CRC patients and assess their clinicopathologic correlations using a practical immunohistochemistry (IHC) panel supplemented by a limited targeted molecular approach. This study included 273 CRC patients diagnosed between 2023 and 2024 at two major referral hospitals in Muscat. Initially, the mismatch repair (MMR)-deficient tumors (dMMR) were assigned as CMS1, while the MMR-proficient (pMMR) tumors were further evaluated for β-catenin, P53, KRAS, and TGF-β expression. Mutations in *BRAF*, *TP53*, and *KRAS* were analyzed by sequencing. The cohort comprised 51.6% males, with a mean age of 59.1 years. Most tumors were left-sided (70.7%). dMMR (CMS1) comprised 31 cases (11.35%). Out of the pMMR tumors, 111 cases (40.65%) showed positive expression of β-catenin and P53 (CMS2), 63 cases (23.0%) showed *KRAS* mutations (CMS3), and 68 cases (24.9%) showed TGF-β-positive expression (CMS4). The cases were predominantly concentrated in Muscat (41%). This study demonstrated the feasibility and clinical relevance of CMS-based classification in Oman and its potential role in precision oncology and healthcare planning.

## 1. Introduction

Colorectal cancer (CRC) represents a substantial and escalating global public health burden. According to the International Agency for Research on Cancer (IARC), more than 1.9 million new CRC cases and approximately 930,000 CRC-related deaths were recorded worldwide in 2020, ranking CRC as the third most commonly diagnosed malignancy and the second leading cause of cancer-related mortality globally [1]. Projections indicate that by 2040, the global incidence may rise to approximately 3.2 million new cases annually, with the number of CRC-related deaths increasing to nearly 1.6 million. This anticipated growth reflects ongoing demographic changes, particularly population expansion and aging [2].

This increasing burden is mirrored in many transitional and developing regions, including the Middle East, where rapid urbanization, shifts toward Westernized dietary patterns, sedentary lifestyles, and aging populations are contributing to the rising incidence of CRC [3]. In Oman, CRC is already among the most frequently diagnosed malignancies. A recent national analysis reported that in 2020, CRC accounted for approximately 10.4% of all newly diagnosed cancers, ranking second only to breast cancer. Furthermore, CRC was identified as the leading cause of cancer-related mortality among males and the second leading cause among females [4]. Longitudinal data demonstrated a steady increase in age-standardized incidence rates (ASRs) of CRC in Oman between 1997 and 2019, with consistently higher rates observed in males, underscoring the urgent need for effective screening, risk stratification, and management strategies [4]. These epidemiological trends highlight a critical public health imperative: beyond conventional screening and prevention, the deeper molecular characterization of CRC in Omani patients may enable more precise diagnostic, prognostic, and therapeutic approaches. Such efforts are particularly relevant given the potential influence of population-specific genetic, environmental, and lifestyle factors.

CRC is a biologically heterogeneous disease. Tumors that appear histologically similar may differ markedly in their molecular drivers, clinical behavior, and response to therapy. To address this complexity, the international research community developed the Consensus Molecular Subtypes (CMS) classification, a biologically grounded framework based on tumor gene expression profiles [5]. In the landmark 2015 study integrating multiple prior classification systems, four distinct CMS groups were defined, each characterized by unique molecular pathways, clinicopathological features, and prognostic implications [5]. CMS1 (MSI-immune) accounts for approximately 14% of CRCs and is characterized by a high mutational burden, microsatellite instability (MSI), frequent BRAF mutations, and prominent immune cell infiltration, reflecting an activated immune microenvironment. These tumors are typically right-sided and exhibit distinct clinical behavior [6]. CMS2 (canonical/epithelial), the most prevalent subtype (~37%), is defined by activation of the WNT and MYC signaling pathways, chromosomal instability with many somatic copy number alterations, and preserved epithelial differentiation. CMS2 tumors are more commonly left-sided and are generally associated with a relatively favorable prognosis [7,8]. CMS3 (metabolic), representing approximately 13% of CRCs, is also epithelial in nature, but distinguished by metabolic dysregulation and a high frequency of activating KRAS mutations. This subtype exhibits mixed microsatellite status and fewer copy number alterations compared with CMS2 [7]. CMS4 (mesenchymal) accounts for roughly 23% of CRCs and is characterized by prominent stromal infiltration, activation of transforming growth factor-β (TGF-β), angiogenesis-related pathways, and features of epithelial–mesenchymal transition (EMT). Clinically, CMS4 tumors are associated with aggressive behavior, increased metastatic potential, and a poor prognosis [9].

Notably, approximately 13% of tumors in the original CMS cohort demonstrated mixed or indeterminate features, reflecting intratumoral heterogeneity or transitional phenotypes. Subsequent studies have established the prognostic and predictive relevance of CMS classification. A recent meta-analysis demonstrated that in early-stage CRC, CMS4 tumors are associated with significantly lower overall survival rates compared with CMS1 and CMS2, while CMS2 and CMS3 derive the greatest benefit from adjuvant chemotherapy in stage II–III disease [6]. In a metastatic setting, CMS classification has been linked to differential responses to targeted therapies. CMS2 tumors may respond more favorably to anti-EGFR agents in the context of RAS wild-type status; CMS1 tumors show enhanced responsiveness to immune checkpoint inhibitors; and CMS4 tumors may exhibit increased sensitivity to irinotecan-based chemotherapy regimens [6]. Collectively, these findings position CMS as a key framework underpinning precision oncology in CRC [7].

Despite its promise, routine clinical implementation of CMS classification remains limited, particularly in resource-constrained settings. The reference standard for CMS assignment relies on transcriptomic profiling techniques, such as RNA sequencing, which require substantial financial investment, specialized laboratory infrastructure, and bioinformatics expertise [8]. To address these challenges, surrogate approaches based on immunohistochemistry (IHC) and limited molecular testing have been developed [10].

An early IHC-based classifier utilized markers such as FRMD6, ZEB1, HTR2B, and CDX2 to approximate the CMS categories; however, this approach struggled to reliably distinguish between CMS2 and CMS3, the two epithelial subtypes. Subsequent refinement incorporating nuclear β-catenin staining, a surrogate for WNT pathway activation, improved discrimination between these subtypes, achieving concordance rates of approximately 71–77% compared with transcriptomic classification in validation cohorts [11]. More recent strategies combine IHC markers (including mismatch repair proteins for MSI/CMS1, β-catenin, and stromal or mesenchymal markers) with limited molecular testing, such as KRAS mutation analysis, offering a pragmatic and cost-effective alternative when comprehensive transcriptomic profiling is not feasible [9]. Nevertheless, important limitations persist, including intratumoral heterogeneity, overlapping phenotypes—particularly between CMS2 and CMS3—and the absence of universally standardized surrogate classification protocols [11].

The main aim of this study is to clearly highlight the CRC consensus subtypes in an Omani cohort and correlate these with clinicopathologic features to bridge the gap in molecular characterization of CRC in Oman and the Gulf region. To the best of our knowledge, no similar studies have been carried out.

In the context of Oman’s rising CRC burden, integrating a CMS-informed framework could offer several advantages. From a prognostic perspective, CMS classification may facilitate risk stratification, enabling identification of high-risk patients, such as those with CMS4 tumors, who may benefit from intensified surveillance [12]. Therapeutically, CMS-based stratification could guide treatment selection, assigning therapies to patients that are most likely to be effective, such as immunotherapy for CMS1 or targeted therapy for CMS2 [13]. From a health system perspective, surrogate CMS classification using IHC and limited molecular testing could optimize resource utilization by providing molecularly informed decision making without prohibitive costs [10]. Finally, characterizing the CMS distribution within the Omani population may yield valuable epidemiological insights, revealing whether the molecular landscape parallels global patterns or reflects population-specific features that warrant tailored management strategies [4].

## 2. Results

Clinical and demographic features: Out of the 273 cases included, 141 (51.6%) were males, and 132 (48.4%) were females. The ages ranged from 24 to 94 years, with a mean age of 59.14 and a standard deviation of 14.43. Tumor distribution by site included the right colon (*n* = 80, 29.3%) and the left colon (*n* = 193, 70.3%). Out of the 273 tumors, 46 (16.85%) were mucinous, and the rest, *n* = 227 (83.15%), were non-mucinous. Table 1 summarizes the clinical and demographic features.

### 2.1. MMR IHC and CMS Assignment

The patient samples were assigned to CMS subtypes based on the criteria outlined below:

CMS1 (MSI-immune): Any case with deficient MMR on IHC was assigned to CMS1. All CMS1 cases underwent BRAF V600E testing by PCR to aid in distinguishing sporadic from hereditary MSI [5].

CMS2 (canonical/epithelial): Proficient MMR cases with nuclear β-catenin and p53 aberrant staining and epithelial morphology were allocated to CMS2.

CMS3 (metabolic): Proficient MMR cases with KRAS mutations predominating and without dominant stromal/TGF-β features were assigned to CMS3.

CMS4 (mesenchymal): Proficient MMR cases showing a prominent stromal reaction, high TGF-β expression levels and mesenchymal features were classified as CMS4 [6,7]

**Figure 1:**
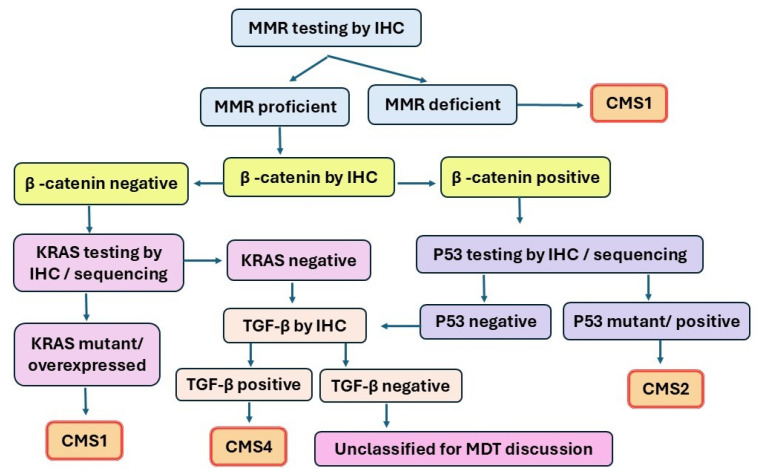
Algorithm for categorizing CMS subtypes, testing, and classification.

The cases that did not clearly fit into a single surrogate category after review were discussed in a multidisciplinary pathology meeting and assigned based on dominant features. The limitations of surrogate classification versus transcriptomic assignment were acknowledged. An algorithm for CMS type assignment is shown in Figure 1.

The distribution of CMS types (Table 2) among the tested patients was determined as follow:

dMMR (*n* = 31, 11.4%): showed loss of expression of one or more MMR proteins and was classified as (CMS1). The remaining 242 cases (88.6%) were proficient for MMR (pMMR). Out of the dMMR cases, only one case showed a *BRAF* V600E mutation, while the remaining 30 cases showed wild-type BRAF. 

**Figure 2:**
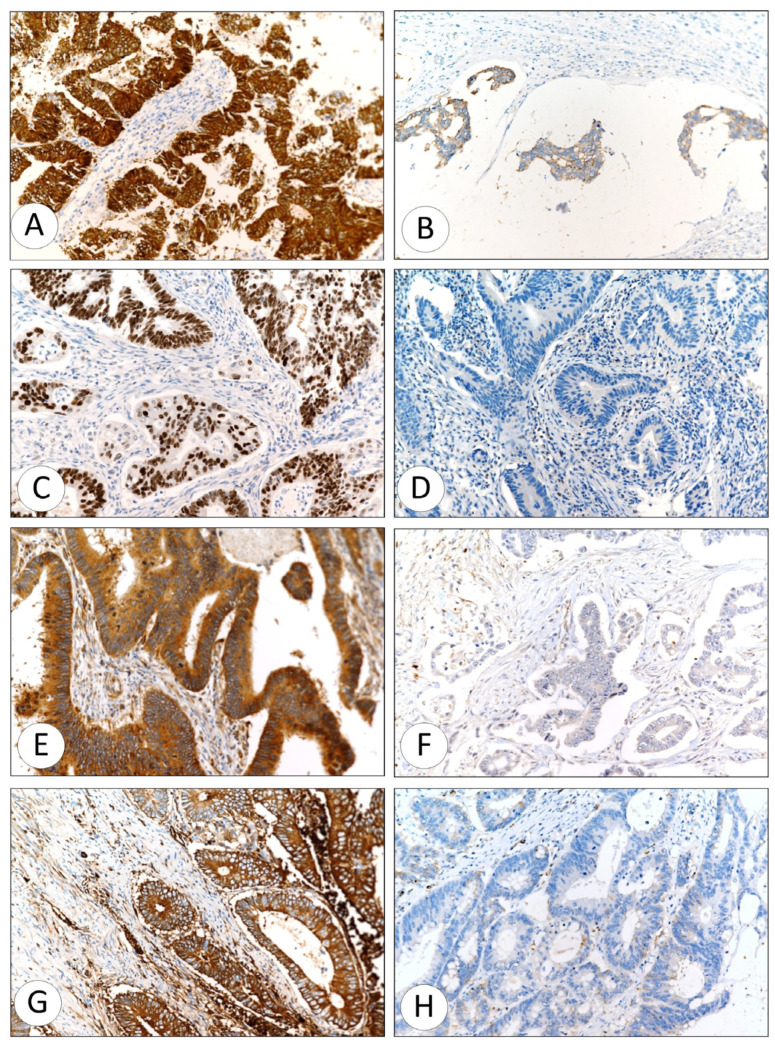
Representative photomicrographs of immune histochemical reactivity to different antibodies used in this study. β-catenin, example of positive reactivity (**A**) and negative reactivity (**B**); P53, positive reactivity (**C**) and negative reactivity (**D**); KRAS, positive reactivity (**E**) and negative reactivity (**F**); and TGF-β, positive reactivity (**G**) and negative reactivity (**H**). All pictures were taken at 20× magnification.

pMMR profiling (*n* = 242 cases, 88.6%):

CMS2 (β-catenin nuclear localization + P53 aberrant): (*n* = 111, 40.6%) (Figure 2A–D).

CMS3 (*KRAS* mutation/overexpression): (*n* = 63, 23.07%) (Figure 2E,F).

CMS4 (TGF-β positive with stromal/mesenchymal features): (*n* = 68, 25%) (Figure 2G,H).

**Table 2:** Distribution of consensus molecular subtypes (CMS) in colorectal cancer cases in Oman (*n* = 273).

CMS Group	Classification Basis	*n* (%)
CMS1	dMMR by IHC	31 (11.40)
CMS2	pMMR; nuclear β-catenin + aberrant p53	111 (40.60)
CMS3	pMMR; KRAS mutation positive	63 (23.00)
CMS4	pMMR; TGF-β expression + stromal/mesenchymal features	68 (25.00)

Notes: Percentages are rounded to one decimal place for consistency. Abbreviations: CMS = consensus molecular subtype; IHC = immunohistochemistry; MMR = mismatch repair; dMMR = deficient MMR; pMMR = proficient MMR.

### 2.2. Correlation of CMS Types with Various Clinical Parameters

A total of 273 CRC cases were included; 141 (51.6%) were males, and 132 (48.4%) were females. CMS2 was the most common subtype in both genders, though it was more prevalent in the females than the males, with 57/132 (43.18%) being females and 54/141 (38.29%) being males, followed by CMS4 in 36/141 (25.50%) males and 32/ 132 (24.24%) females.

Tumors were predominantly located in the left colon (193/273; 70.7%) compared with the right colon (80/273; 29.3%). Among the molecular subtypes, CMS1 was most commonly observed in the right colon (18/31; 58.06%), and CMS2 was most commonly observed in the left colon (81/111; 72.97%). A significant association was found between the CMS subtypes and tumor stage (*p* = 0.001), histology status (*p* < 0.001), tumor location (*p* = 0.002), and pathology T stage (*p* < 0.001). In contrast, no significant associations were found between age (*p* = 0.704), gender (*p* = 0.449), and grade (*p* = 0.061). Table 3 summarizes the correlation between the CMS types and the clinical parameters. 

### 2.3. Correlation of CRC Cases and CMS Distribution with Different Regions of Oman

**Figure 3:**
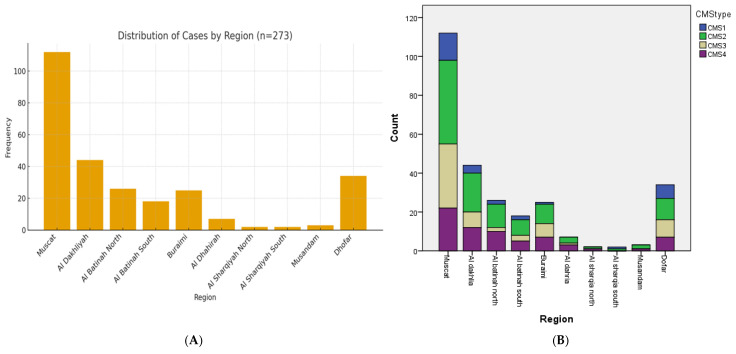
(**A**): Distribution of CRC cases in different regions of Oman. (**B**): Distribution of CMS subtypes across different regions of Oman.

The geographical distribution of 273 colorectal cancer cases across various regions of Oman showed an apparent regional disparity, with Muscat accounting for the highest proportion of cases (*n* = 112, 41%), representing the main contributor to the overall cohort. The second-highest burden was seen in Al Dakhiliyah (*n* = 44, 16% cases), followed by Al-Batinah North and Buraimi, each contributing around *n* = 27, 24 respectively. Al Batinah South shows a slightly lower frequency (*n* = 18 cases), indicating moderate representation from this region. In contrast, several regions display relatively low case numbers. Al Dhahirah, Al Sharqiyah North, Al Sharqiyah South, and Musandam each contribute fewer than 10 cases. Dhofar stands out within the southern governorates, contributing 34 cases, which is higher than several northern regions. The geographic distribution of cases across regions is shown in Figure 3A.

The regional distribution of the Consensus Molecular Subtypes (CMS1–CMS4) among colorectal cancer (CRC) cases across Oman showed an overall pattern that closely mirrors the distribution of CRC cases, with Muscat accounting for the most significant proportion across all the CMS subtypes. Among the Muscat cases, CMS2 and CMS3 appear prominent, followed by CMS4 and CMS1, reflecting the molecular heterogeneity typically observed in larger, more diagnostically active regions. Al Dakhiliyah has the second-highest number of cases and represents all four CMS subtypes. Similar to Muscat, CMS2 accounts for the majority of cases, with smaller, but noticeable contributions from CMS3 and CMS4, and fewer CMS1 cases. This distribution underscores the substantial CRC burden and diagnostic activity in this region. The distribution of different CMS types in the different areas of Oman is shown in Figure 3B.

## 3. Discussion

The CMS classification by Guinney et al. [5] remains the canonical reference for describing the molecular, biological, and clinical features of the four CMS groups: CMS1 (MSI-immune), CMS2 (canonical/WNT/MYC), CMS3 (metabolic/KRAS-enriched), and CMS4 (mesenchymal/TGF-β/stromal). Our study employed this framework and adapted a pragmatic IHC/targeted molecular testing approach as a surrogate algorithm to allocate cases to a specific CMS subtype [11].

In this series of 273 colorectal adenocarcinomas from two tertiary hospitals in Muscat, Oman, we utilized a panel of IHC markers, along with selected targeted molecular markers, to classify the patients according to the CMS types. This study assigned the cases to the CMS groups as follows: CMS1 (*n* = 31, 11.4%), CMS2 (*n* = 111, 40.6%), CMS3 (*n* = 63, 23.0%), and CMS4 (*n* = 68, 25.0%). The predominance of canonical/epithelial (CMS2) tumors and the proportion of mesenchymal (CMS4) tumors are the two most salient features.

Most of the large, studied cohorts of CRC patients show variable CMS distributions; however, in concordance with our findings, CMS2 was reported as the most typical type in many studies, as reported by Kheirelseid et al. [13] and Guiney et al. [5].

The predominance of CMS2 type in our cohort as well as in other studies reflects the nature of this type as it follows the classic adenoma–carcinoma sequence initiated through APC inactivation with Wnt/β-catenin activation and progresses through chromosomal instability with *TP53* mutations. It is affected also by environmental drivers such as diet, obesity and smoking. It also represents the most “treatable” CRC subtype with the current standard therapies [13].

The proportion of CMS1 (MSI) in population-based series varies widely by geography, screening practices, and patient age; many studies report MSI/dMMR proportions of ~10–20% overall [14,15].

Our observed dMMR frequency (11.4%) aligns with these reports [16]. However, our dMMR cases showed a very low frequency of BRAF V600E in contrast to many western series where sporadic MSI cases due to MLH1 methylation often harbor this *BRAF* mutation. The low *BRAF* frequency in our cohort necessitates further testing for MLH1 promoter methylation studies as well as germline MSI testing. This differentiation has a great impact on patient management regarding genetic counselling, surveillance and screening for extracolonic malignancies. *BRAF* mutant tumors tend to have a worse overall prognosis, but they open the door to BRAF-targeted therapies, which are not applicable to wild-type *BRAF* tumors.

This difference could either reflect population differences in the proportion of Lynch syndrome versus sporadic MSI, or technical and sampling differences. This can be solved by systematic MLH1 promoter methylation testing and germline assessment to uncover the distribution of sporadic versus hereditary MSI CRC cases in Oman, although the cases with wild-type BRAF were analyzed for MSI mutations (Lynch syndrome screening), and all were found to be negative for the specific MSI tests [17].

The representation of CMS3 (KRAS-enriched) in our cohort (23%) is somewhat lower than in other molecular datasets, which report a higher proportion of KRAS-mutant tumors [18]. It is essential to note that KRAS mutations are heterogeneous, and the detection methods (molecular sequencing vs. surrogate IHC) and the selection criteria used influenced the previously reported frequencies [18]. Recent studies indicate that KRAS mutations are enriched in CMS3, and this influences the tumor immune microenvironment and response to therapy. This finding supports the clinical relevance of identifying this subgroup [19].

The relatively high proportion of CMS4 (25%) in this Omani cohort in comparison to the previous studies warrants further emphasis [20]. CMS4 is linked to mesenchymal/stromal gene expression, TGF-β pathway activation, angiogenesis, and a poor prognosis; it is also associated with resistance to specific therapies and increased rates of metastasis and recurrence in several studies. The high CMS4 fraction could reflect case mixing (e.g., referral bias with more advanced or aggressive tumors at tertiary centers), biological differences in the regional population, or limitations of the surrogate IHC assignment [21], which can increase the chance of CMS4 diagnosis when stroma is prominent in biopsy samples. Further transcriptomic profiling and correlation with clinical outcomes (disease-free and overall survival) would clarify the prognostic weight of this local observation.

The MS1 cases (dMMR) were more frequently right-sided (proximal) in our cohort, consistent with prior literature describing a predilection of MSI tumors for the proximal colon. CMS 2 and CMS2 were more commonly found on the left side [22].

Survival and prognosis of the patients will be monitored as all the patients were newly recruited in a period over the last two years. A follow up study will be designed to link the reported CMS types to prognosis and survival.

### Clinical and Translational Implications

The CMS framework has potential clinical utility to guide health policies as follows: CMS1 (dMMR/MSI-immune) is a likely candidate for immune checkpoint blockade, and so identification via MMR IHC is a cost-effective strategy to screen patients for immunotherapy eligibility and to trigger Lynch syndrome evaluation [23]. Our finding of 11.4% dMMR supports routine universal MMR testing in CRC in Oman. The low *BRAF* mutation frequency in our cohort of dMMR suggests hereditary rather than sporadic origin and necessitates further testing for MLH1 promoter methylation studies, as well as germline MSI testing. This differentiation has a great impact on patient management regarding genetic counselling, surveillance and screening for extracolonic malignancies. *BRAF* mutant tumors tend to have a worse overall prognosis, but they open the door to BRAF-targeted therapies, which are not applicable to wild-type BRAF tumors.

The dominant CMS2 (WNT/β-catenin) and p53 alterations canonically support the therapeutic strategies targeting the WNT pathway or vulnerabilities linked to epithelial signaling and may encourage clinical trials on this pathway.

The *KRAS* mutations in CMS3 (metabolic/KRAS enriched) denote limited benefit from anti-EGFR therapies in advanced disease and may direct enrolment in KRAS-targeted trials where available. Given the association of CMS4 (mesenchymal) type with poor prognosis and stromal-driven biology (TGF-β), CMS4 tumors may be treating using approaches targeting the stroma, TGF-β signaling, or angiogenesis, though clinical trial evidence is evolving. Patients with CMS4 disease may warrant closer surveillance, earlier systemic therapy, or trial enrollment despite having similar TNM stages. The high local prevalence suggests a need for prioritized research into the biology and management of CMS4 in Oman [24,25].

This high prevalence of CMS4 may be related to underlying genetic changes in the Omani population. The current limited, generic approach we followed may also require further IHC and genetic testing for better discrimination of this category. The lack of long-term clinical outcome data, including those on disease-free and overall survival, precluded direct assessment of the prognostic impact of CMS subtypes in this cohort.

From a health policy perspective, establishing pathology workflows that include universal MMR IHC and selective targeted molecular and IHC testing (KRAS, BRAF, and p53/β-catenin when indicated) is feasible in many tertiary settings, providing there is actionable information for both prognostication and treatment.

For example, patients with the CMS1 subtype (MSI-high, immune-rich tumors) are strong responders to immune checkpoint inhibitors. Patients with the CMS2 subtype have potentially benefit more from using anti-EGFR agents to treat RAS wild-type tumors, while patients with the CMS3 subtype (KRAS-driven) showed a limited benefit from EGFR inhibitors. Patients with the CMS4 subtype with angiogenesis and TGF-β signaling activation may require combination strategies (e.g., immunotherapy and TGF-β inhibition) and may benefit from anti-VEGF therapies and novel stromal-targeting agents. CMS4 tumors show aggressive local invasion and a higher recurrence risk, which may influence decisions on neoadjuvant therapy, wider surgical margins and more aggressive management of oligometastatic disease. Based on these findings, integrating surrogate CMS assignment into multidisciplinary tumor boards could help personalize therapy and guide clinical trial selection until transcriptomic profiling becomes more readily available [26].

Finally, we propose the following algorithm to implicate the CMS types in clinical practice: (i) universal MMR immunohistochemistry to identify CMS1; (ii) selective targeted molecular testing (KRAS and BRAF) combined with surrogate IHC markers (β-catenin, p53) to enable provisional CMS assignment for CMS2–4; and (iii) incorporation of CMS information into multidisciplinary tumor board discussions for therapeutic stratification. Clearly linking CMS classification to diagnostic workflows will help in treatment selection, research prioritization, and health policy planning.

## 4. Materials and Methods

### 4.1. Study Design and Setting

This is a prospective cross-sectional, hospital-based study of consecutive CRC cases diagnosed between January 2023 and December 2024 at the Gastroenterology outpatient departments of Sultan Qaboos University Hospital (SQUH) and the Royal Hospital (RH) in Muscat, Oman. The pathology services of SQUH acted as the central laboratory for immunohistochemical and molecular testing.

### 4.2. Sample Selection and Data Collection

All consecutive patients who presented to the gastroenterology outpatient clinics at the SQUH and the RH with suspected CRC and underwent colonoscopic biopsy or surgical resection with histopathologically confirmed colorectal adenocarcinoma during the study period were included. Patients diagnosed with other malignancies, such as neuroendocrine tumors, lymphomas, and gastrointestinal stromal tumors, were excluded from this study. Patients with inadequate tissue for IHC/DNA extraction and patients who received neoadjuvant therapy were also excluded. Two hundred seventy-three (*n* = 273, 168 from the RH and 105 from SQUH) eligible cases were included. Two representative H&E slides of each case were retrieved and reviewed. Then, the allocated paraffin-fixed tissue blocks were selected that showed both tumor and adjacent non-tumor colonic epithelium. The cases were re-evaluated and confirmed for histological type and grade. The clinical data were collected from hospital information systems.

All the procedures were approved by the Ethics Committee of Sultan Qaboos University [Institutional Review Board (IRB) Ref. REF. NO. SQU-EC/251\2023 MREC #3239 dated 7 March 2024] and in agreement with the Declaration of Helsinki of 1964 and its later amendments. Written informed consent was obtained from each participant prior to this study.

### 4.3. Tissue Preparation and IHC Protocol

#### 4.3.1. Tissue Microarray Preparation

Tissue microarrays (TMAs) were prepared from the tissue blocks using the Manual Tissue Arrayer MTA-1 from Estigen OU, Tiigi 61b, 50410 Tartu, Estonia. Finally, 273 TMA paraffin blocks were prepared, each containing malignant and control cylindrical tissue cores arranged according to a pre-prepared map.

#### 4.3.2. Immunohistochemistry

Tissue microarray sections were obtained at a thickness of 4 µm and stained immunohistochemically by an automated stainer (BenchMark, Roche, Tucson, AZ, USA), with antibodies against a panel of antibodies including MMR proteins (MLH1, MSH2, MSH6, and PMS2), β-catenin, P53, TGF-β and wild-type KRAS antibodies. The details and conditions for each antibody are shown in Table 4.

Adjacent normal colonic epithelium, lymphocytes, and stromal cells served as positive internal controls. Negative controls were done by replacing the primary antibody with PBS. The procedures were performed according to the manufacturer’s instructions.

#### 4.3.3. Evaluation of Immunohistochemical Reactivity

The stained slides were evaluated by two pathologists (AQ and AS) with no prior knowledge of the patient details and outcomes using standard light microscopes.

Interpretation of mismatch repair protein expression

The staining intensity and distribution of each protein (MSH2, MSH6, MLH1, and PMS2) were assessed, and the results were classified into two categories: negative (loss of expression), indicating no nuclear immune reactivity; and positive, indicating any positive nuclear immune reactivity in the neoplastic cells. The patient was considered MMR-deficient when one or more of the four tested proteins showed lost expression [12].

Interpretation of P53, β-catenin, TGF-β and KRAS:

P53 was scored as ‘aberrant’ (diffuse intense nuclear staining or complete absence) versus the ‘wild-type’ pattern. β-catenin was classified as membranous/cytoplasmic or nuclear, and only nuclear expression was considered positive. TGF-β was graded as absent/weak versus moderate/strong stromal and/or tumor cell expression. KRAS staining was interpreted as follows: negative or weak membranous staining/cytoplasmic blush (negative), intermediate bright staining or intense cytoplasmic staining (positive) [13].

### 4.4. Molecular Testing (DNA Extraction, PCR/Sequencing)

#### 4.4.1. DNA Extraction and Molecular Testing

For each of the tested tumors, 2 mm diameter tumor tissue cylinders were punched from formalin-fixed, paraffin-embedded (FFPE) tissue blocks and collected in an Eppendorf tube. Genomic DNA was isolated from FFPE tissue block using the GeneJET FFPE DNA purification kit (Thermo Fisher Scientific, Waltham, MA, USA/EEC). DNA quantification was performed by spectrophotometry (Nanodrop—Biodrop Resolution, Cambridge, UK).

PCR testing was performed using end-point PCR for *BRAF* G469 on exon 11 and V600 on exon 15; *KRAS* G12 and G13 on exon 2; and Q61 on exon 3; and *TP53* R175 on exon 5; G245 and R248 on exon 7; and R273 and R282 on exon 8. The primers used and the PCR conditions are summarized in Table 5.

Sixty nanograms of DNA was used for PCR amplification using the TrueAllele PCR Premix kit (Thermo Fisher Scientific, Waltham, MA, USA/EEC) and verified on a 1% agarose gel for proper amplification. Amplicons were then purified using the ExoSAP-IT PCR Product Cleanup kit (Thermo Fisher Scientific, Waltham, MA, USA/EEC).

Sanger sequencing was performed on purified amplicons using the BigDye Terminator v3.1 Cycle Sequencing Kit (Thermo Fisher Scientific, Waltham, MA, USA/EEC). Forward or reverse primers used in the end-point PCR were used in respective sequencing reactions (Table 5). Details of the PCR conditions and sequencing reaction program are provided in Supplementary Tables S1 and S2. Post-sequencing processing was performed using 0.125 mM EDTA, 96–100% molecular-grade ethanol, and 70% molecular-grade ethanol (Sigma, St. Louis, MO, USA/EEC). Sequenced samples were resuspended in Hi-Di Formamide (Thermo Fisher Scientific, Waltham, MA, USA/EEC) and analyzed on a 3500 Genetic Analyzer (Thermo Fisher Scientific, Waltham, MA, USA/EEC). Chromatograms were manually inspected using the BioEdit 7.7 (https://thalljiscience.github.io/ URL accessed 30 September 2024) and Chromas (Technelysium Pty Ltd., Soth Brisbane, QLD, Australia) programs.

#### 4.4.2. Statistical Analysis

Descriptive statistics (frequencies and percentages) summarized CMS distribution, sex, age, and tumor site. Continuous variables are presented as mean ± standard deviation (SD) or the median (interquartile range), depending on the distribution. Where relevant, χ^2^ or Fisher’s exact tests compared the CMS subtypes with the categorical variables (e.g., tumor site). *p*-value < 0.05 was considered statistically significant. Analyses were performed using IBM SPSS v25. Because this study was observational and descriptive, no a priori sample size calculation was performed [27]. Ethical approval was obtained from the institutional review boards of SQUH and the Royal Hospital, and consent procedures followed institutional standards for use of archival tissue.

#### 4.4.3. Strengths and Limitations

This study represents the first comprehensive report on CMS-like stratification in an Omani CRC cohort using centrally performed IHC and targeted molecular marker testing. The rise of centralized laboratory procedures and dual pathologist review are the key strengths that enhances this study’s consistency.

On the other hand, several limitations should be acknowledged. First, transcriptomic profiling—the current gold standard for CMS classification—was not performed, which may have led to partial misclassification of tumors, particularly those with mixed or ambiguous phenotypes. Second, as a hospital-based study from two tertiary centers, the cohort may overrepresent advanced or aggressive cases, limiting generalizability to the wider Omani population. Third, clinical outcome data (e.g., recurrence, survival, and treatment response) were not yet available, precluding direct evaluation of prognostic or predictive implications. Finally, despite central review, minor interobserver variation in IHC interpretation cannot be completely excluded.

#### 4.4.4. Recommendations for Future Work

The results of this study support the utmost significance of CRC screening in Oman, based on the finding that CMS2 is the most prevalent in this Omani cohort. This type is known to be familial in origin and linked to the WNT FAP pathway. People who are diagnosed with this type of cancer should have the whole family screened for familial cancers. Additionally, CMS1 accounts for approximately 11% and may also be a component of Lynch syndrome. This also needs probing and familial screening. There is a strong need for a national multicenter registry to assess the geographic and ethnic differences in patients of CRC within Oman and the wider Gulf region.

We should consider cost-effectiveness analyses to guide the adoption of routine MMR/KRAS/BRAF testing and selective transcriptomic profiling in national practice.

## 5. Conclusions

Applying consensus molecular subtype concepts to colorectal cancers in Oman using an IHC and targeted molecular marker surrogate algorithm identified CMS2 and CMS4 as the dominant groups, with a meaningful minority of CMS1 (dMMR) and CMS3 (KRAS-driven) tumors. These findings support universal MMR testing and targeted molecular profiling in routine diagnostics, underscoring the need for further molecular and outcome-linked research to guide precision oncology strategies tailored to the Omani population.

## Figures and Tables

**Table 1:** Clinical and demographic features of colorectal cancer patients (*n* = 273).

Variable	*n* (%)
Age group	
<50 years	72 (26.4%)
≥50 years	201 (73.6%)
Gender	
Male	141 (51.6%)
Female	132 (48.4%)
Tumor site	
Right colon	80 (29.3%)
Left colon	193 (70.7%)
Histologic type	
Mucinous	46 (16.9%)
Non-mucinous	227 (83.1%)
Tumor stage	
I	27 (9.9%)
II	100 (36.6%)
III	128 (46.9%)
IV	18 (6.6%)

**Table 3:** Summary of the correlation between CMS types and the clinical parameters (*n* = 273).

Clinicopathological Variable		CMS1 *n* (%)	CMS2 *n* (%)	CMS3 *n* (%)	CMS4 *n* (%)	*p*-Value
		31 (11.30)	111 (40.02)	63 (23.07)	68 (24.90)	
Age						
<50		9 (29.04)	31 (27.92)	13 (20.63)	19 (27.95)	0.704 ^a^
≥50		22 (70.96)	80 (72.08)	50 (79.37)	49 (72.05)	
Sex						
Female		11 (35.48)	57 (51.35)	32 (50.79)	32 (47.06)	0.449 ^a^
Male		20 (64.52)	54 (48.65)	31 (49.21)	36 (52.94)	
Grade						
Low		29 (93.55)	93 (83.78)	50 (79.36)	49 (72.05)	0.061 ^a^
High		2 (6.45)	18 (16.22)	13 (20.64%)	19 (27.95)	
Tumor location						
Right colon		18 (58.06)	31 (27.92)	16 (25.39)	15 (22.05)	0.002 ^a^
Left colon		13 (41.94)	80 (72.08)	47 (74.61)	53 (77.95)	
Histology						
Non-mucinous		16 (51.61)	109 (98.19)	52 (82.54)	50 (73.52)	<0.001 ^a^
Mucinous		15 (48.31)	2 (1.81)	11 (17.46)	18 (26.48)	
Depth of tumor invasion						
T1	*n* = 6	1 (16.66)	4 (66.68)	1 (16.66)	0 (0.00)	<0.001 ^b^
T2	*n* = 23	6 (26.10)	7 (30.40)	9 (39.10)	1 (4.40)	
T3	*n* = 172	23 (13.38)	66 (38.38)	33 (19.18)	50 (29.06)	
T4	*n* = 72	1 (1.38)	34 (47.23)	20 (27.77)	17 (23.62)	
Tumor stage						
Stage I	*n* = 27	7 (25.94)	10 (37.03)	9 (33.33)	1 (3.70)	0.001 ^b^
Stage I	*n* = 100	18 (18.00)	35 (35.00)	18 (18.00)	29 (29.00)	
Stage III	*n* = 128	5 (3.90)	57 (44.54)	32 (25.00)	34 (26.56)	
Stage IV	*n* = 18	1 (5.55)	9 (50.00)	4 (22.23)	4 (22.22)	

Notes: Data are presented as *n* (%). ^a^ χ^2^ test; ^b^ Fisher’s exact test (Monte Carlo). Abbreviation: CMS = consensus molecular subtype.

**Table 4:** Details of the antibodies used for IHC.

	Antibody	Company	Type	Clone	Antigen Retrieval	Dilution
1	MLH1	Ventana, Tucson, AZ, USA	Mouse Monoclonal	M1	64 min	Ready to use
2	MSH2	Ventana, Tucson, AZ, USA	Mouse Monoclonal	G219-1129	64 min	Ready to use
3	MSH6	Ventana, Tucson, AZ, USA	Mouse Monoclonal	SP93	64 min	Ready to use
4	PMS2	Ventana, Tucson, AZ, USA	Rabbit Monoclonal	A16 4 [EPR3947]	92 min	Ready to use
5	β-catenin	Ventana, Tucson, AZ, USA	Mouse Monoclonal	14	64 min	Ready to use
6	P53	Ventana, Tucson, AZ, USA	Mouse Monoclonal	PB53-11	64 min	Ready to use
7	TGF beta	Abcam, Cambridge UK	Rabbit monoclonal	TB21	64 min	1:200
8	KRAS	Novus, Chesterfield, MO, USA	Rabbit monoclonal	NBP3-03659 [11]	64 min	1:100

**Table 5:** Primer sequences used in PCR amplification and sequencing reactions.

Primer		Forward	Reverse
*BRAF*	G469	5′-TGATTGGGAGATTCCTGATGGG-3′	5′-TGATGCGAACAGTGAATATTTCCT-3′
	V600	5′-TGCTTGCTCTGATAGGAAAATG-3′	5′-CCACAAAATGGATCCAGACA-3′
	G12 G13	5′-AAGGCCTGCTGAAAATGAC-3′	5′-TGGTCCTGCACCAGTAATATG-3′
*KRAS*	Q61	5′-CCAGACTGTGTTTCTCCCTTCT-3′	5′-CCCTCCCCAGTCCTCATGTA-3′
	R175	5′-GTGCAGCTGTGGGTTGATTC-3′	5′-TCAGTGAGGAATCAGAGGCC-3′
*TP53*	G245 R248	5′-CCACAGGTCTCCCCAAGG-3′	5′-CAGCAGGCCAGTGTGCAG-3′
	R273 R282	5′-GCCTCTTGCTTCTCTTTTCC-3′	5′-TAACTGCACCCTTGGTCTCC-3′

## Data Availability

The original contributions presented in this study are included in this article/Supplementary Material. Further inquiries can be directed to the corresponding author.

## Supplementary Materials

The following supporting information can be downloaded at: https://www.mdpi.com/article/10.3390/ijms27042038/s1.

## References

[1] Matsuda T., Fujimoto A., Igarashi Y. (2025). Colorectal Cancer: Epidemiology, Risk Factors, and Public Health Strategies. Digestion.

[2] Keum N., Giovannucci E. (2019). Global burden of colorectal cancer: Emerging trends, risk factors and prevention strategies. Nat. Rev. Gastroenterol. Hepatol..

[3] Zhou J., Yang Q., Zhao S., Sun L., Li R., Wang J., Wang L., Wang D. (2025). Evolving landscape of colorectal cancer: Global and regional burden, risk factor dynamics, and future scenarios (the Global Burden of Disease 1990–2050). Ageing Res. Rev..

[4] Kumar S., Burney I.A., Zahid K.F., Souza P.C.D., Belushi M.A.L., Mufti T.D., Meki W.A., Furrukh M., Moundhri M.S. (2015). Colorectal Cancer Patient Characteristics, Treatment and Survival in Oman—A Single Center Study. Asian Pac. J. Cancer Prev..

[5] Guinney J., Dienstmann R., Wang X., de Reyniès A., Schlicker A., Soneson C., Marisa L., Roepman P., Nyamundanda G., Angelino P. (2015). The consensus molecular subtypes of colorectal cancer. Nat. Med..

[6] Soldevilla B., Carretero-Puche C., Gomez-Lopez G., Al-Shahrour F., Riesco M.C., Gil-Calderon B., Alvarez-Vallina L., Espinosa-Olarte P., Gomez-Esteves G., Rubio-Cuesta B. (2019). The correlation between immune subtypes and consensus molecular subtypes in colorectal cancer identifies novel tumour microenvironment profiles, with prognostic and therapeutic implications. Eur. J. Cancer.

[7] Rebersek M. (2020). Consensus molecular subtypes (CMS) in metastatic colorectal cancer—Personalized medicine decision. Radiol. Oncol..

[8] Valdeolivas A., Amberg B., Giroud N., Richardson M., Gálvez E.J.C., Badillo S., Julien-Laferrière A., Túrós D., Voith von Voithenberg L., Wells I. (2024). Profiling the heterogeneity of colorectal cancer consensus molecular subtypes using spatial transcriptomics. NPJ Precis. Oncol..

[9] Li Y., Yao Q., Zhang L., Mo S., Cai S., Huang D., Peng J. (2020). Immunohistochemistry-Based Consensus Molecular Subtypes as a Prognostic and Predictive Biomarker for Adjuvant Chemotherapy in Patients with Stage II Colorectal Cancer. Oncologist.

[10] Kantha A., Das D., Pai E., Kumar T., Pandey M. (2025). Consensus Molecular Subtypes (CMS) Classification: A progress towards Subtype-Driven treatments in colorectal cancer. World J. Surg. Oncol..

[11] Lenz H.J., Ou F.S., Venook A.P., Hochster H.S., Niedzwiecki D., Goldberg R.M., Mayer R.J., Bertagnolli M.M., Blanke C.D., Zemla T. (2019). Impact of Consensus Molecular Subtype on Survival in Patients With Metastatic Colorectal Cancer: Results From CALGB/SWOG 80405 (Alliance). J. Clin. Oncol..

[12] Kheirelseid E.A., Miller N., Chang K.H., Curran C., Hennessey E., Sheehan M., Kerin M.J. (2013). Mismatch repair protein expression in colorectal cancer. J. Gastrointest. Oncol..

[13] Dvorak K., Higgins A., Palting J., Cohen M., Brunhoeber P. (2019). Immunohistochemistry with Anti-BRAF V600E (VE1) Mouse Monoclonal Antibody is a Sensitive Method for Detection of the BRAF V600E Mutation in Colon Cancer: Evaluation of 120 Cases with and without KRAS Mutation and Literature Review. Pathol. Oncol. Res..

[14] Khaliq A.M., Erdogan C., Kurt Z., Turgut S.S., Grunvald M.W., Rand T., Khare S., Borgia J.A., Hayden D.M., Pappas S.G. (2022). Refining colorectal cancer classification and clinical stratification through a single-cell atlas. Genome Biol..

[15] Dienstmann R., Vermeulen L., Guinney J., Kopetz S., Tejpar S., Tabernero J. (2017). Consensus molecular subtypes and the evolution of precision medicine in colorectal cancer. Nat. Rev. Cancer.

[16] Song N., Pogue-Geile K.L., Gavin P.G., Yothers G., Kim S.R., Johnson N.L., Lipchik C., Allegra C.J., Petrelli N.J., O’Connell M.J. (2016). Clinical Outcome From Oxaliplatin Treatment in Stage II/III Colon Cancer According to Intrinsic Subtypes. JAMA Oncol..

[17] Parsons M.T., Buchanan D.D., Thompson B., Young J.P., Spurdle A.B. (2012). Correlation of tumour BRAF mutations and MLH1 methylation with germline mismatch repair (MMR) gene mutation status: A literature review assessing utility of tumour features for MMR variant classification. J. Med. Genet..

[18] Tanjak P., Chaiboonchoe A., Suwatthanarak T., Acharayothin O., Thanormjit K., Chanthercrob J., Suwatthanarak T., Wannasuphaphol B., Chumchuen K., Suktitipat B. (2023). The KRAS-Mutant Consensus Molecular Subtype 3 Reveals an Immunosuppressive Tumor Microenvironment in Colorectal Cancer. Cancers.

[19] Lal N., White B.S., Goussous G., Pickles O., Mason M.J., Beggs A.D., Taniere P., Willcox B.E., Guinney J., Middleton G.W. (2018). KRAS Mutation and Consensus Molecular Subtypes 2 and 3 Are Independently Associated with Reduced Immune Infiltration and Reactivity in Colorectal Cancer. Clin. Cancer Res..

[20] Herrera M., Berral-González A., López-Cade I., Galindo-Pumariño C., Bueno-Fortes S., Martín-Merino M., Carrato A., Ocaña A., De La Pinta C., López-Alfonso A. (2021). Cancer-associated fibroblast-derived gene signatures determine prognosis in colon cancer patients. Mol. Cancer.

[21] Valenzuela G., Canepa J., Simonetti C., Solo de Zaldívar L., Marcelain K., González-Montero J. (2021). Consensus molecular subtypes of colorectal cancer in clinical practice: A translational approach. World J. Clin. Oncol..

[22] Sinicrope F.A., Shi Q., Smyrk T.C., Thibodeau S.N., Dienstmann R., Guinney J., Bot B.M., Tejpar S., Delorenzi M., Goldberg R.M. (2015). Molecular Markers Identify Subtypes of Stage III Colon Cancer Associated With Patient Outcomes. Gastroenterology.

[23] Okita A., Takahashi S., Ouchi K., Inoue M., Watanabe M., Endo M., Honda H., Yamada Y., Ishioka C. (2018). Consensus molecular subtypes classification of colorectal cancer as a predictive factor for chemotherapeutic efficacy against metastatic colorectal cancer. Oncotarget.

[24] Trinh A., Trumpi K., De Sousa EMelo F., Wang X., de Jong J.H., Fessler E., Kuppen P.J., Reimers M.S., Swets M., Koopman M. (2017). Practical and Robust Identification of Molecular Subtypes in Colorectal Cancer by Immunohistochemistry. Clin. Cancer Res..

[25] de Back T.R., Wu T., Schafrat P.J., ten Hoorn S., Tan M., He L., van Hooff S.R., Koster J., Nijman L.E., Vink G.R. (2024). A consensus molecular subtypes classification strategy for clinical colorectal cancer tissues. Life Sci. Alliance.

[26] Guo S.B., Hu L.S., Huang W.J., Zhou Z.Z., Luo H.Y., Tian X.P. (2024). Comparative investigation of neoadjuvant immunotherapy versus adjuvant immunotherapy in perioperative patients with cancer: A global-scale, cross-sectional, and large-sample informatics study. Int. J. Surg..

[27] Kim H.Y. (2017). Statistical notes for clinical researchers: Chi-squared test and Fisher’s exact test. Restor. Dent. Endod..

