# Correction: Rehman et al. Analysis of Consensus Molecular Subtypes of Colorectal Cancer in Oman with Clinicopathologic Correlation. *Int. J. Mol. Sci.* 2026, *27*, 2038

**DOI:** 10.3390/ijms27062578

**Published:** 2026-03-12

**Authors:** Shaista Rehman, Asem Shalaby, Said A. Al-Busafi, Moon Fai Chan, Shaima Al Khabouri, Mustafa Al Hinai, Adhari Al Zaabi, Mohammad Al Masqari, Asim Qureshi, Mohammed Al-Azri

**Affiliations:** 1 Department of Family Medicine and Public Health, College of Medicine and Health Sciences, Sultan Qaboos University, Muscat 123, Oman; s139506@student.squ.edu.om (S.R.); moonf@squ.edu.om (M.F.C.); mus0031@squ.edu.om (M.A.H.); mhalazri@squ.edu.om (M.A.-A.); 2 Department of Pathology, College of Medicine and Health Sciences, Sultan Qaboos University, Muscat 123, Oman; a.qureshi@squ.edu.om; 3 Department of Pathology, College of Medicine, Mansoura University, Mansoura 1156, Egypt; 4 Gastroenterology and Hepatology Unit, Department of Medicine, College of Medicine and Health Sciences, Sultan Qaboos University, Muscat 123, Oman; busafis@squ.edu.om; 5 Medical Research Center, Sultan Qaboos University, Muscat 123, Oman; 6 Department of Medicine, Faculty of Medicine and Health, Sepuluh Nopember Institute of Technology, Surabaya 60111, Indonesia; 7 Department of Biochemistry, College of Medicine and Health Sciences, Sultan Qaboos University, Muscat 123, Oman; s.alkhabouri@squ.edu.om; 8 Human and Clinical Anatomy Department, College of Medicine and Health Sciences, Sultan Qaboos University, Muscat 123, Oman; adhari@squ.edu.om; 9 Department of Pathology, The Royal Hospital, Ministry of Health, Muscat 113, Oman; mme9090@gmail.com

## Additional Affiliations

In the published publication [1], there was an error regarding the affiliations of Said A. Al-Busafi and Moon Fai Chan. In addition to affiliation 4, the updated affiliation should include the following: Medical Research Center, Sultan Qaboos University, Muscat 123, Oman (Said A. Al-Busafi). In addition to affiliation 1, the updated affiliation should include the following: Department of Medicine, Faculty of Medicine and Health, Sepuluh Nopember Institute of Technology, Surabaya 60111, Indonesia (Moon Fai Chan).

The authors state that the scientific conclusions are unaffected. This correction was approved by the Academic Editor. The original publication has also been updated.
