# Supplementary material for: Analysis of Consensus Molecular Subtypes of Colorectal Cancer in Oman with Clinicopathologic Correlation
Source: Int J Mol Sci. 2026 Feb 21;27(4):2038. doi: 10.3390/ijms27042038 (PMC12940371; doi:10.3390/ijms27042038)
Supplement: Supplementary file 1 [file ijms-27-02038-s001.zip › ijms-4075491-supplementary.pdf]

Table S1 End point PCR program

	Stage 1	Stage 2			Stage 3	
**Step**	Hot start activation	Denaturation	Annealing	Extension	Final extension	Hold
**Temp**	95°C	95°C	60°C	72°C	72°C	4°C
**Time**	5 min	30 sec	30 sec	1 min	7 min	infinity
**No. Cycles**	1X	40X			1X	

Table S2 BigDye Terminator v3.1 sequencing program

	Stage 1	Stage 2			Stage 3
**Step**	Initial denaturation	Denaturation	Annealing	Extension	Hold
**Temp**	96°C	96°C	50°C	60°C	4°C
**Time**	1 min	10 sec	5 sec	4 min	infinity
**No. Cycles**	1X	35X			1X
